# GM101 in Combination with Histone Deacetylase Inhibitor Enhances Anti-Tumor Effects in Desmoplastic Microenvironment

**DOI:** 10.3390/cells10112811

**Published:** 2021-10-20

**Authors:** Han-Gyu Chang, Yong-Hyeon Choi, JinWoo Hong, Joung-Woo Choi, A-Rum Yoon, Chae-Ok Yun

**Affiliations:** 1 Department of Bioengineering, College of Engineering, Hanyang University, 222 Wangsimni-ro, Seongdong-gu, Seoul 04763, Korea; charley26@hanyang.ac.kr (H.-G.C.); chois0802@gmail.com (J.-W.C.); 2 GeneMedicine CO., Ltd., 222 Wangsimni-ro, Seongdong-gu, Seoul 04763, Korea; yhchoi@gene-medicine.com (Y.-H.C.); jhong803@gene-medicine.com (J.H.); 3 Institute of Nano Science and Technology (INST), Hanyang University, 222 Wangsimni-ro, Seongdong-gu, Seoul 04763, Korea

**Keywords:** GM101, histone deacetylase inhibitor, MS-275, SBHA, CAR receptor, dynamin, clathrin, relaxin

## Abstract

Oncolytic adenoviruses (oAds) have been evaluated in numerous clinical trials due to their promising attributes as cancer therapeutics. However, the therapeutic efficacy of oAds was limited due to variable coxsackie and adenovirus receptor (CAR) expression levels and the dense extracellular matrix (ECM) of heterogenic clinical tumors. To overcome these limitations, our present report investigated the therapeutic efficacy of combining GM101, an oAd with excellent tumor ECM degrading properties, and histone deacetylase inhibitor (HDACi). Four different HDACi (suberohydroxamic acid (SBHA), MS-275, trichostatin A (TSA), and valproic acid) candidates in combination with replication-incompetent and GFP-expressing Ad (dAd/GFP) revealed that SBHA and MS-275 exerted more potent enhancement in Ad transduction efficacy than TSA or valproic acid. Further characterization revealed that SBHA and MS-275 effectively upregulated CAR expression in cancer cells, improved the binding of Ad with cancer cell membranes, and led to dynamin 2- and clathrin-mediated endocytosis of Ad. The combination of GM101 with HDACi induced superior cancer cell killing effects compared to any of the monotherapies, without any additional cytotoxicity in normal cell lines. Further, GM101+SBHA and GM101+MS-275 induced more potent antitumor efficacy than any monotherapy in U343 xenograft tumor model. Potent antitumor efficacy was achieved via the combination of GM101 with HDACi, inducing necrotic and apoptotic cancer cell death, inhibiting cancer cell proliferation, degrading ECM in tumor tissue, and thus exerting the highest level of virus dispersion and accumulation. Collectively, these data demonstrate that the combination of GM101 and HDACi can enhance intratumoral dispersion and accumulation of oAd through multifaced mechanisms, making it a promising strategy to address the challenges toward successful clinical development of oAd.

## 1. Introduction

In eukaryotic cells, histone acetyltransferase and histone deacetylase (HDAC) play a crucial role in the regulation of DNA transcription and gene expression through the addition and removal of acetyl groups on the lysine residues of histones [1]. The HDAC-mediated gene regulation process is dysregulated and promotes oncogenic phenotype of cancer cells, thus the inhibition of HDAC with chemical inhibitors (HDACi) is under active preclinical and clinical investigation with several products, such as vorinostat (suberanilohydroxamic acid) and panobinostat (hydroxamic acid), approved by the US Food and Drug Administration [2,3,4,5]. HDACi treatment has been shown to reactivate tumor suppressor genes or death signaling pathways [6] and induce apoptosis of cancer cells [7], resulting in antitumor efficacy.

Currently, oncolytic adenoviruses (oAds) are under clinical evaluation across multiple clinical trials to treat various types of cancer [8,9,10]. oAds induce highly preferential killing of cancer cells through cancer-specific replication of the virus and exponential amplification of therapeutic transgene expression, thus resulting in antitumor effects [11]. Further, oAd has minimal insertional mutagenesis risks and a well-documented safety record across multiple clinical trials being conducted from the 1990s [12].

Despite the first oncolytic Ad being commercialized in China as early as 2005, no Ad-based therapeutic has been approved for clinical use in Europe or the USA to date, due to inadequate therapeutic benefits. Subsequent investigations have revealed that insufficient tumoral infection of oAd is one of the limitations contributing to suboptimal therapeutic efficacy in clinics. In detail, variable expression of the coxsackie-adenovirus receptor (CAR) in heterogenic tumor populations and ablation of CAR expression with disease progression in a subset of tumor cases can severely hamper CAR-mediated infection and the spreading of the adenovirus (Ad) across the tumor tissues [13,14,15,16]. To this end, several groups have reported that HDACi can be an attractive combination therapy candidate for oAd, since HDACi has been shown to increase CAR expression levels of cancer cells [17], enhance the transcription of oAd genome in target cells [18], sensitize tumor cells toward induction of apoptosis [19,20,21,22], and ultimately augment the cytolytic and antitumor effects of oAd [23,24].

A dense extracellular matrix (ECM) of the tumor microenvironment has also been identified as a key barrier to efficient intratumoral dispersion of oAd throughout the entirety of tumor milieu [25]. To address this challenge, the present report has investigated the combination of GM101, a modified TERT-regulated oAd that expresses human relaxin and contains the wild-type fiber of human serotype 5 Ad [26,27], and HDACi. Relaxin is a peptide hormone that is structurally related to insulin and insulin-related growth factors [28]. In previous studies, the relaxin treatment in lung fibrosis (i.e., the common end stage of many pneumopathies) animal model decreased the synthesis and secretion of interstitial collagens and increased the expression of matrix metalloproteinase and procollagenase [29]. Through the combination of GM101 and HDACi, we aimed to maximize the therapeutic value through enhancement of intratumoral dispersion of the virus via upregulating intratumoral CAR expression levels and degrading the aberrant tumor ECM, respectively.

Based on these backgrounds, the present study evaluated several different HDACi (suberohydroxamic acid (SBHA), MS-275, trichostatin A (TSA), and valproic acid) in combination with Ad to screen for the optimal combination therapy candidates. Of the four HDACi, SBHA and MS-275 were found to synergistically enhance the potency of Ad. Our findings demonstrate that both HDACi (SBHA and MS-275) enhance the internalization of Ad into cancer cells by upregulating key endocytic proteins, such as CAR, dynamin 2, and clathrin. Importantly, our findings demonstrate that the combination of HDACi with GM101 exert more potent antitumor effects than monotherapies by inducing robust ECM degradation and viral replication in tumor tissues.

## 2. Materials and Methods

### 2.1. Cell Culture and Preparation of Ad

Human lung cancer cell line (A549), human glioblastoma (GBM) cell line (U343), normal fibroblast cell lines (CBHEL, BJ), normal brain astroglia cell line (SVG p12), and normal lung fibroblast cell line (IMR-90) were purchased from the American Tissue Culture Collection (Manassas, VA, USA). A549, U343, CBHEL, SVG p12, and IMR-90 cells were maintained in Dulbecco’s Modified Eagle’s Medium (DMEM; Pan biotech, Aidenbach, Germany). BJ cells were maintained in Minimum Essential Medium (Hyclon, Logan, UT, USA). Authentication of U343 cell line by short tandem repeat analysis was conducted by Cosmogenetech (Seoul, Korea). All culture mediums were supplemented with 10% fetal bovine serum (FBS; Alpha bioregen, Boston, MA, USA). All cells were grown in an incubator at 37 °C with 5% CO_2_. Detailed construction and generation of green fluorescence protein (GFP)-expressing replication-incompetent Ad (dAd/GFP) [11], replication-incompetent Ad expressing IX protein tagged with GFP in fusion form (dAd/IX-GFP) [30], and GM101 [31,32] have been described previously. The schematic representation of the virus genome structure of GM101 can be found in Figure S1.

### 2.2. Preparation of HDACi

MS-275 was purchased from Alexis Biochemicals (Lausen, Switzerland). SBHA, TSA, and valproic acid were purchased from Sigma-Aldrich (St. Louis, MO, USA). All HDACi were dissolved in dimethyl sulfoxide (DMSO; Sigma-Aldrich) then serially diluted in phosphate buffered saline (PBS) prior to usage.

### 2.3. Transduction Efficiency Assay

Transduction efficiency was assessed by quantifying GFP expression in U343 and A549 cells. Cells were seeded at a density of 1 × 10^4^ cells/well in 48-well plates for 24 h. The cells were treated with SBHA (1–20 μM for U343 and 1–100 μM for A549) or MS-275 (0.1–2 μM) for 24 h, along with PBS as a negative control. Following HDACi pretreatment, cells were transduced with dAd/GFP at a multiplicity of infection (MOI) of 10 (U343) or 5 (A549). At 48 h after transduction, the cells were observed and quantified for GFP using the IncuCyte^®^ Live-Cell Analysis System (Sartorius, Goettingen, Germany).

### 2.4. Quantification of CAR Expression Level

CAR expression levels of cancer cells were determined by flow cytometry. U343 and A549 cells were treated with SBHA (20 μM) or MS-275 (2 μM) for 24 h, along with PBS as a negative control. Cells were incubated with a fluorescein isothiocyanate (FITC)-linked anti-CAR antibody (Ab; Santa Cruz, Dallas, TX, USA) for 20 min, harvested, then resuspended in FACS buffer (PBS containing 0.5% bovine serum albumin (Millipore, Billerica, MA, USA), 0.05% sodium azide (Sigma-Aldrich), and 1 mM EDTA (Sigma-Aldrich)). Samples were analyzed by FACS Calibur analyzer (BD Bioscience, San Jose, CA, USA) using CellQuest software (BD Bioscience). Data from 10,000 cell events were collected and analyzed for each sample.

### 2.5. Total Internal Reflection Fluorescence (TIRF) Microscope Imaging

TIRF microscopy was performed with a Nikon Eclipse TE2000 inverted fluorescence microscope (Nikon, Tokyo, Japan) equipped with a 488 nm argon ion laser (Coherent, Santa Clara, CA, USA) and a digital camera (Hamanatsu, Bridgewater, NJ, USA). The microscope was fitted with a CFI apochromat oil-immersion objective lens (Nikon) (100F, NA = 1.49). Fluorescence signals were detected through a long-pass dichroic mirror (>505 nm) and a band-pass filter (510–560 nm). For live cell imaging, U343 cells were seeded onto custom-made glass bottom dishes (MaTek cultureware, Ashland, MA, USA), and grown to approximately 10–20% confluence. Cells were incubated with SBHA (20 μM) or MS-275 (2 μM) for 24 h. Then, cells were treated with dAd/IX-GFP (500 MOI) and images were captured every 2 min for a total of 20 m with exposure time of 1 ms. The filter set and the camera were controlled by Metamorph software (Universal Imaging, Downingtown, PA, USA).

### 2.6. Analysis of Virus Attachment to Cell Membrane Following HDACi Treatment

GM101 was labeled with FITC (Sigma-Aldrich) for 2 h at room temperature to generate GM101-FITC, then dialyzed (10 kDa MWCO, Slide-A-Lyzer™ Dialysis Cassettes, Thermo Fisher Scientific, Waltham, MA, USA) overnight in ice-cold 10 mM Tris to remove unreacted FITC. U343 and A549 cells were treated with PBS, SBHA (20 μM for U343 and 100 μM for A549 cells, respectively) or MS-275 (2 μM) for 24 h then resuspended in FACS buffer at the cell density of 2 × 10*^5^* cells/sample. Subsequently, the cells were prechilled at 4 °C for 30 min then treated with 1,000 MOI of GM101-FITC at 4 °C for 2 h under constant shaking to allow for virus attachment to the cell membranes. The cell pellets were washed 3 times with FACS buffer to remove any unbound GM101-FITC then fixed in 1% paraformaldehyde solution in PBS. Samples were analyzed by FACS Calibur analyzer as described above in Section 2.4.

### 2.7. Analysis of Clathrin- and Dynamin-Dependent Endocytosis of Ad Following HDACi Treatment

U343 cells were pretreated with dynamin 2 inhibitor (dynasore—40 μM, Enzo Life Sciences, Farmingdale, NY, USA), clathrin inhibitor (CPZ—5 μM, Enzo Life Sciences), or caveolin inhibitor (genistein—37 μM, Enzo Life Sciences) for 45 min, along with PBS as a negative control. Then the cells were treated with SBHA (20 μM) or MS-275 (2 μM) for 24 h, along with PBS as a negative control. Subsequently, the cells were transduced with dAd/GFP at an MOI of 50 or 100 for another 24 h. The GFP expression was analyzed and quantified using the IncuCyte^®^ Live-Cell Analysis System (Sartorius) as described above.

### 2.8. Western Blot Analysis

Cells were treated with SBHA (20 μM) or MS-275 (2 μM) for 24 h. Cells were lysed in NP-40 (Elpisbio, Daejeon, Korea) in the presence of a protease inhibitor cocktail (Sigma-Aldrich). Protein concentration was determined using the bicinchoninic acid protein assay (Pierce Rockford, IL Waltham, MA, USA). Each sample was separated by sodium dodecyl sulfate-polyacrylamide gel electrophoresis (SDS-PAGE) and electroblotted onto a polyvinyl difluoride (PVDF) membrane. Membranes were incubated with anti-dynamin-2 (BD Bioscience), anti-clathrin (Abcam, Cambridge, UK), and anti-β-actin (Santa Cruz) Abs then incubated with either horseradish peroxidase (HRP)-conjugated anti-rabbit IgG (Cell signaling technology, Beverly, MA, USA) or HRP-conjugated anti-mouse IgG (Cell signaling technology) secondary Abs. Blots were visualized using a super-signal chemiluminescence kit (Pierce, Rockford, IL, USA) and developed using image quant LAS 4000 (GE Lifesciences, Pittsburgh, PA, USA).

### 2.9. Cell Viability Assessment

To evaluate the cancer cell-specific killing effect of the different monotherapies and combination therapies, cancer cells (U343 or A549) or normal cells (CBHEL, BJ, IMR-90, or SVG p12) were seeded at 1 × 10^4^ cells/well on a 24-well plate and treated with various concentrations of SBHA or MS-275 for 24 h, along with PBS as a negative control. For the combination therapy groups, HDACi-treated cells were infected with 10 MOI of GM101, whereas the negative control group and HDACi monotherapy groups were treated with PBS. At 48 h post infection, 250 μL of 3-(4,5-dimethylthiazol-2-yl)-2,5-diphenyltetrazolium bromide (MTT; Sigma-Aldrich) reagent dissolved in PBS at a final concentration of 2 mg/mL was added to each well and incubated at 37 °C for 4 h. Then the supernatant was discarded, and the precipitated formazan was dissolved in 1 mL of DMSO (Sigma-Aldrich). Optical density of the dissolved formazan product was read on a microplate reader at 540 nm (Spectramax Mw; Molecular devices, Sunny Vale, CA, USA). The optical density readings from the PBS-treated wells were considered to be 100% viable.

### 2.10. Assessment of Antitumor Efficacy

To evaluate the antitumor efficacy of different treatments, a U343 human xenograft tumor model was established subcutaneously by injecting 1 × 10^7^ cells under the abdominal skin of 6- to 8-week-old male athymic nude mice (Orientbio, Seong-nam, Korea). Once the average tumor volumes reached 100 mm^3^, mice were randomized into six groups (PBS, SBHA, MS-275, GM101, GM101+SBHA, and GM101+MS-275) to begin treatments (designated as day 1). The mice were intraperitoneally injected with PBS, SBHA (200 mg/kg), or MS-275 (20 mg/kg) on day 1, 3, and 5. PBS or GM101 (5 × 10^9^ VPs) were intratumorally administered on days 2, 4, and 6. The length (L) and width (W) of the tumor were measured every other day with a caliper to calculate tumor growth. The tumor volume was calculated according to the following formula: tumor volume = 0.523 × L × W^2^. All animal studies were conducted under the institutional guidelines of Hanyang University Institutional Animal Care and Use Committee.

### 2.11. Histological and Immunohistochemical Analysis of Tumor Tissues

For histological analysis, U343 tumor-bearing mice were treated in the same manner as described above. The tumor tissues were harvested on day 9, embedded in paraffin (Leica, Wetzlar, Germany), and then sectioned at a thickness of 5 μm. Section slides were stained with hematoxylin and eosin (H & E, Sigma-Aldrich) as described in the manufacturer’s instruction.

For immunohistochemical staining, the tumor section slides were blocked with 3% bovine serum albumin in Tris-buffered saline (Sigma-Aldrich) for 2 h. For detection of Ad E1A or proliferating cell nuclear antigen (PCNA), the slides were incubated with a rabbit anti-Ad E1A polyclonal (Santa Cruz) or a mouse anti-PCNA Abs (Dako, Glostrup, Denmark) as primary Abs, then reacted with an HRP-conjugated goat anti-rabbit IgG (Cell signaling technology) or an HRP-conjugated goat anti-mouse IgG Ab (Cell signaling technology) as secondary Abs. For the detection of ECM components, tumor slides were incubated with mouse anti-fibronectin monoclonal (Santa Cruz) or goat anti-collagen I polyclonal (Santa Cruz) primary Abs. Secondary Abs were used as described above. Terminal deoxynucleotidyl transferase dUTP nick end labeling (TUNEL) (Merck, Darmstadt, Germany) staining of tumor section slides were performed as described in the manufacturer’s instruction. After incubation of secondary Abs, color was developed using diaminobenzidine/hydrogen peroxidase (Dako) as the chromogen substrate. All immunohistochemically analyzed tumor slides were counterstained with Meyer’s hematoxylin (Sigma-Aldrich) and examined by light microscopy (Axioskop 40; Carl Zeiss, Oberkochen, Germany).

### 2.12. Serum Toxicity Profile of Combination Therapy

To evaluate the serum toxicity profile of combination therapy regimens, an A549 human xenograft tumor model was established by subcutaneously injecting 1 × 10^7^ cells into 6- to 8-week-old male athymic nude mice (Orientbio). Mice were randomized into three groups (PBS, GM101+SBHA, and GM101+MS-275) and treated in the same manner as described in Section 2.10. At 3 days after the last virus injection, mice were sacrificed then the serum was collected. The level of aspartate aminotransferase (AST), alanine aminotransferase (ALT), blood urea nitrogen (BUN), and creatinine (CRE) in serum samples were analyzed by DRI-CHEM NX700 chemistry analyzer (FUJIFILM, Tokyo, Japan)

### 2.13. Statistical Analysis

All these analyses were performed using the two-tailed Student *t*-test or one-way ANOVA (Prism 8.3 software; GraphPad Software, San Diego, CA, USA). Data are expressed as the mean ± SD. *P* values less than 0.05 were considered to be statistically significant.

## 3. Results

### 3.1. HDACi Enhances Ad-Mediated Transgene Expression

**Figure 1:**
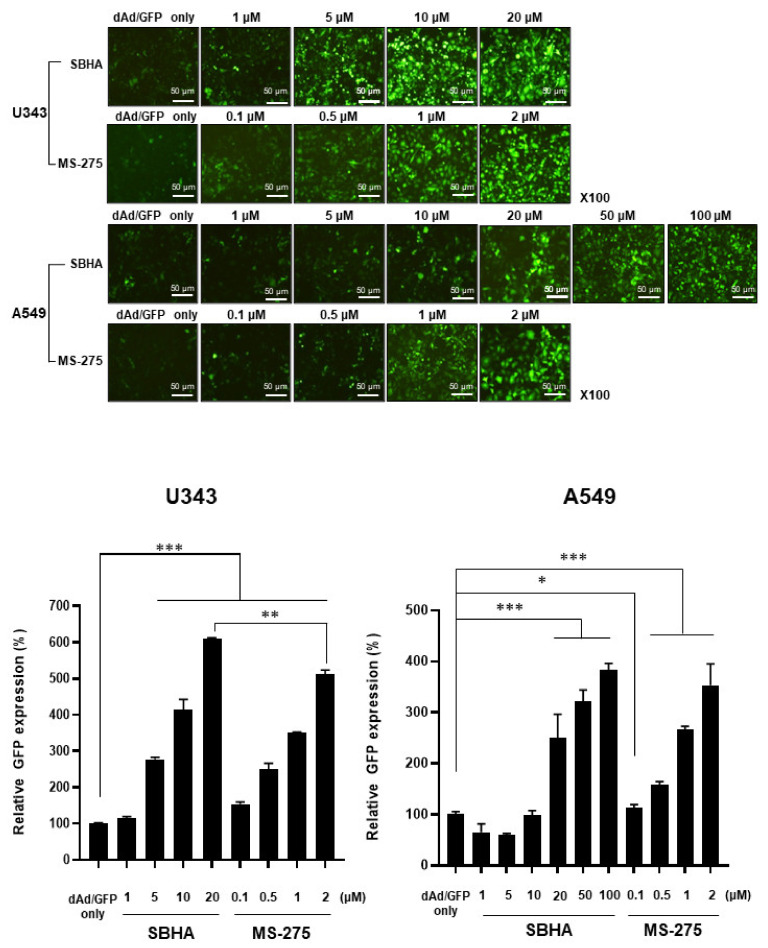
Transduction efficiency of dAd/GFP in combination with HDACi. U343 and A549 cells were pre-treated with SBHA (1–20 μM for U343, 1–100 μM for A549), MS-275 (0.1–2 μM), or PBS for 24 h then transduced with dAd/GFP. At 48 h post transduction, cells were analyzed for GFP expression using the IncuCyte^®^ Live-Cell Analysis System. Representative imaging results are shown. The data are representative of three independent experiments performed in triplicate. Bars represent the mean ± SD. Original magnification: ×100. The scale bar represents 50 µm. ^*^ *P* < 0.05, ^**^ *P* < 0.01, ^***^ *P* < 0.001.

HDACi have been reported to facilitate the internalization of Ad. As there are numerous HDACi in development for cancer therapy, we first investigated which of the HDACi (SBHA, MS-275, TSA, or valproic acid) could enhance the Ad-mediated transgene expression in human cancer cells (U343 and A549). As shown in Figure 1 and Figure S3, pretreatment of cancer cells with any of the four HDACi enhanced the Ad-mediated GFP expression in an HDACi concentration-dependent manner. Of the four HDACi candidates, TSA induced visible cell damage and death at a relatively low dose of 500 nM, while valproic acid required a higher concentration (5–10 mM) to enhance GFP expression levels to a similar level as those achieved with a much lower concentration of the other three HDACi. Based on these findings, SBHA and MS-275 were chosen for the following experiments.

### 3.2. HDACi Upregulates CAR Expression and Facilitates Membrane Attachment of Ad

**Figure 2:**
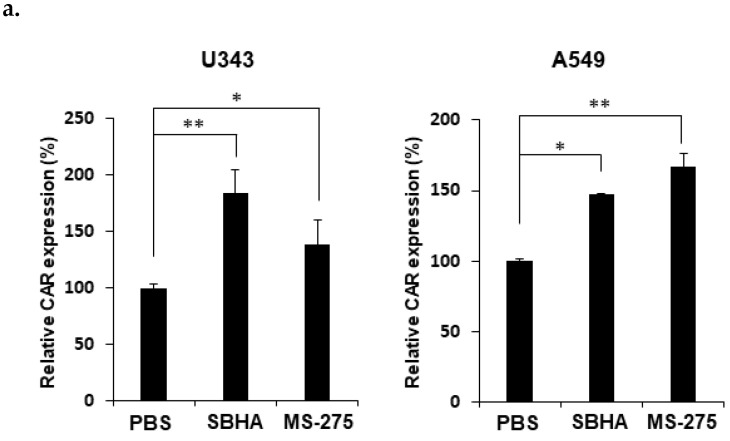

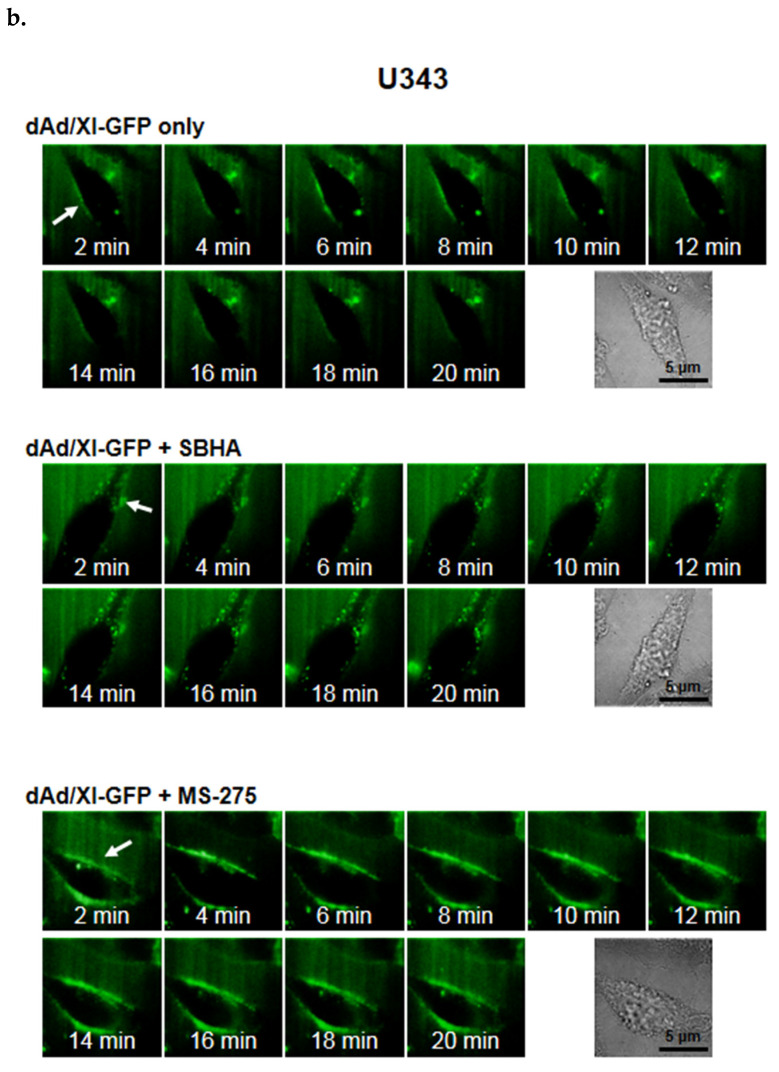
CAR expression level and plasma membrane binding efficiency of Ad following HDACi treatment: (**a**) U343 and A549 cells were treated with either SBHA (20 μM) or MS-275 (2 μM) for 24 h. Cells were analyzed for CAR expression by flow cytometry. The data are representative of three independent experiments performed in triplicate. Bars represent the mean ± SD. ^*^ *P* < 0.05, ^**^ *P* < 0.01. (**b**) U343 cells were pre-treated with SBHA, MS-275, or PBS for 24 h then treated with dAd/IX-GFP. Right after transduction, total internal reflection fluorescence (TIRF) microscope images were captured every 2 min for a total of 20 min with an exposure time of 1 ms to detect Ad accumulation on the plasma membrane of cells. The data are representative of three independent experiments performed in triplicate, and the representative images are shown. The scale bar represents 5 µm.

To evaluate how both SBHA and MS-275 affected the CAR expression level of cancer cell lines, we conducted flow cytometry to assess the CAR expression in U343 and A549 treated with SBHA or MS-275. As shown in Figure 2a and Figure S4, both SBHA and MS-275 treatment significantly increased the CAR expression level in U343 and A549 cells (*P* < 0.05 or 0.01). These results suggest that the CAR upregulating capability of each HDACi in U343 and A549 cell lines contributed to the enhancement of Ad transduction efficacy shown in Figure 1. To further investigate whether the upregulation of CAR by HDACi treatment increases Ad attachment to cancer cells, after U343 cells were treated with dAd/IX-GFP, which has GFP protein tagged to the virus capsid protein IX [30], real-time analysis was conducted by TIRF microscope imaging. As shown in Figure 2b, increased GFP accumulation around the cell membrane was observed in all treatment groups, indicating higher quantities of dAd/IX-GFP bound to the cell surface in the combination therapy groups (dAd/IX-GFP+SBHA and dAd/IX-GFP+MS-275) compared to dAd/IX-GFP-treated group. Similar results were observed when membrane binding of GM101-FITC to PBS- or HDACi-pretreated A549 and U343 cells were analyzed by flow cytometry (Figure S5). Pretreatment of HDACi (SBHA or MS-275) increased the quantity of membrane bound GM101-FITC in both cancer cell lines compared to those bound in PBS-pretreated cells (*P* < 0.05, *P* < 0.01 or *P* < 0.001). Together, these results demonstrate that HDACi-induced elevation of surface CAR expression levels led to improved virus attachment to the cell membrane.

### 3.3. HDACi Induces Cell Uptake of Ad through Dynamin 2- and Clathrin-Mediated Endocytic Pathway

Ads have been reported to internalize into cells via clathrin-mediated endocytosis, which is regulated by dynamin [33,34,35]. Thus, to better elucidate the mechanism behind Ad endocytosis into cells pretreated with HDACi, U343 cells were pretreated with different endocytosis inhibitors: (1) dynasore: a dynamin-mediated endocytosis inhibitor; (2) CPZ: a clathrin-mediated endocytosis inhibitor; or (3) genistein: a caveolae-mediated endocytosis inhibitor. Since the transduction efficiency of dAd/GFP was significantly lower than dAd/GFP+HDACi combination groups, we used 100 MOI of Ad for dAd/GFP only groups and 50 MOI of Ad for dAd/GFP+HDACi combination groups to compensate the low transduction efficiency of naked Ad.

**Figure 3:**
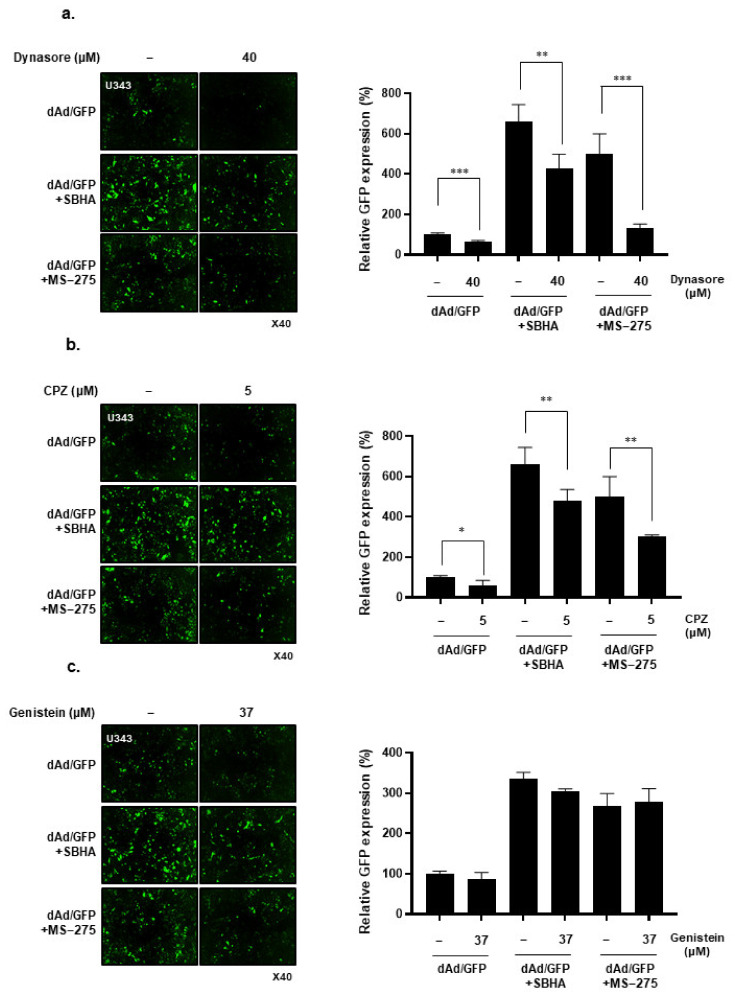
Endocytosis mechanism of Ad in combination with HDACi: (**a**) U343 cells were pretreated with (**a**) dynamin 2 inhibitor (dynasore—40 μM), (**b**) clathrin inhibitor (CPZ—5 μM), or (**c**) caveolin inhibitor (genistein—37 μM) for 45 min. Subsequently, the cells were treated with dAd/GFP, dAd/GFP+SBHA, or dAd/GFP+MS-275. At 24 h post transduction, cells were analyzed for GFP expression by using the IncuCyte^®^ Live-Cell Analysis System (Sartorius). The data are representatives of three independent experiments performed in triplicate. Representative images are shown. Original magnification: ×40. Bars represent the mean ± SD. ^*^ *P* < 0.05, ^**^ *P* < 0.01, ^***^ *P* < 0.001.

As shown in Figure 3a, dynasore pretreatment decreased GFP expression in U343 cells transduced with dAd/GFP (*P* < 0.001), confirming Ad’s dependence on dynamin-mediated endocytosis for cell entry. A decrease of Ad transduction after dynasore treatment was also observed in dAd/GFP+SBHA (*P* < 0.01) or dAd/GFP+MS-275 (P < 0.001) groups, suggesting that the membrane binding and subsequent therapeutic effect of Ad in HDACi- pretreated cells are also depended on dynamin-mediated endocytosis. Reduction of GFP expression after CPZ pretreatment was also observed in all treatment groups (dAd/GFP, *P* < 0.05; dAd/GFP+SBHA, *P* < 0.01; and dAd/GFP+MS-275; *P* < 0.01), showing clathrin-mediated endocytosis of both Ad and Ad+HDACi (Figure 3b). Through further analysis, we found that both HDACi upregulates dynamin 2 and clathrin expression (Figure S6). Together, these results imply that HDACi-induced increases in the expression level of dynamin 2 and clathrin by HDACi may enhance Ad-mediated endocytosis. In contrast, pretreatment of genistein had no inhibitory effect on SBHA- or MS-275-induced enhancement of dAd/GFP transgene expression (Figure 3c), indicating that none of these treatments use caveolin-mediated endocytosis for cellular uptake. Collectively, these results demonstrate that the endocytic cellular uptake of Ad pretreated by HDACi was mostly mediated through dynamin- and clathrin-dependent endocytic pathways.

### 3.4. HDACi Enhances Cytotoxicity of GM101 in a Cancer-Specific Manner

**Figure 4:**
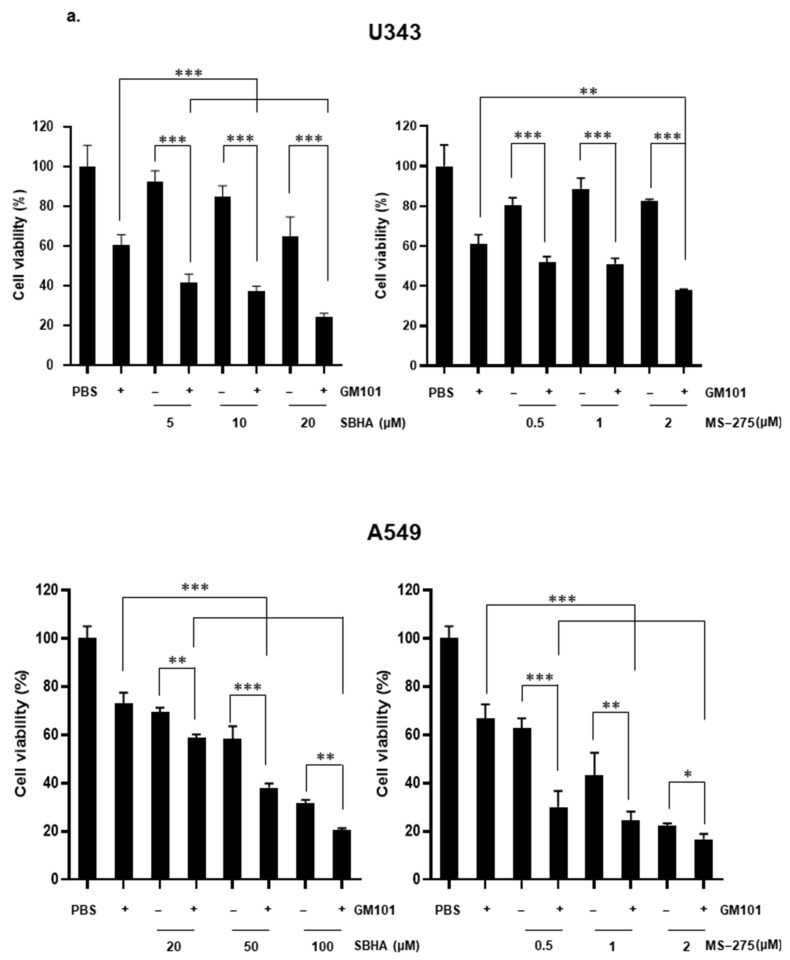

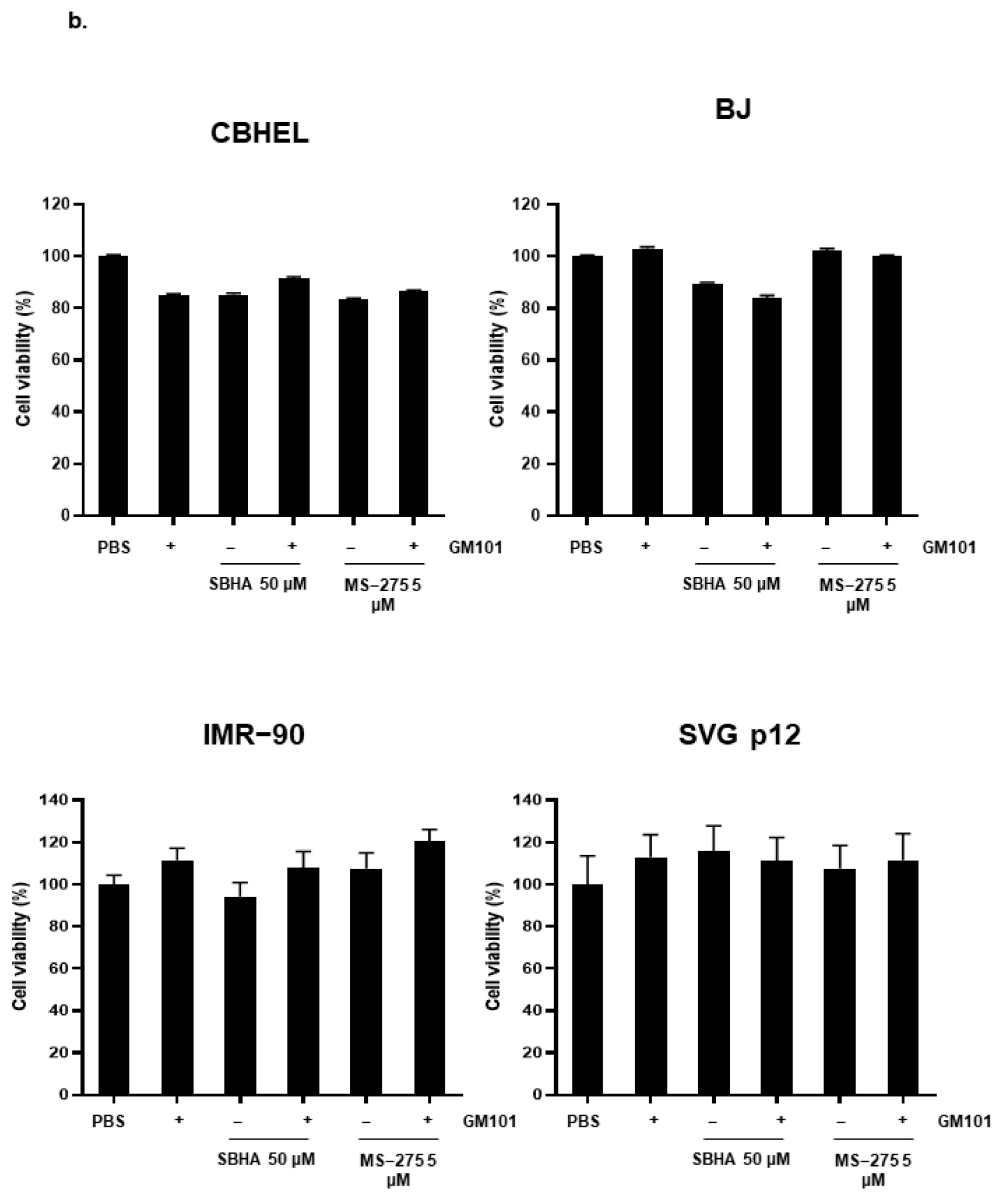
Cancer-specific killing effect of GM101 in combination with HDACi: (**a**) Cancer cells (U343 or A549) and (**b**) normal cells (CBHEL, BJ, IMR-90, or SVG p12) were treated with GM101, GM101+ SBHA, or GM101+MS-275. At 48 h after the GM101 infection, the cell viability was determined by MTT assay. The data are representative of three independent experiments performed in triplicate. Bars represent the mean ± SD. ^*^ *P* < 0.05, ^**^ *P* < 0.01, ^***^ *P* < 0.001.

To evaluate whether HDACi synergizes with oAd in a cancer-specific manner, cancer cells (U343 and A549) and normal cells (CBHEL, BJ, IMR-90, and SVG p12) were treated with GM101 alone or in combination with HDACi (GM101+SBHA or GM101+MS-275). As shown in Figure 4a, GM101+SBHA or GM101+MS-275 treatment elicited significantly more potent cancer cell killing effects than either the GM101 or respective HDACi monotherapy in both U343 and A549 cancer cells (*P* < 0.05, 0.01, or 0.001). In sharp contrast, the GM101+SBHA or GM101+MS-275 treatment elicited a similarly low level of cell killing effects as GM101 or HDACi monotherapy in normal cell lines (Figure 4b). Together, these results demonstrate that HDACi enhances the potency of GM101 in a cancer-specific manner and do not impede the excellent cancer specificity of GM101, thus resulting in a potent and safe combination therapy regimen.

### 3.5. HDACi Potentiate the Antitumor Effect of GM101

**Figure 5:**
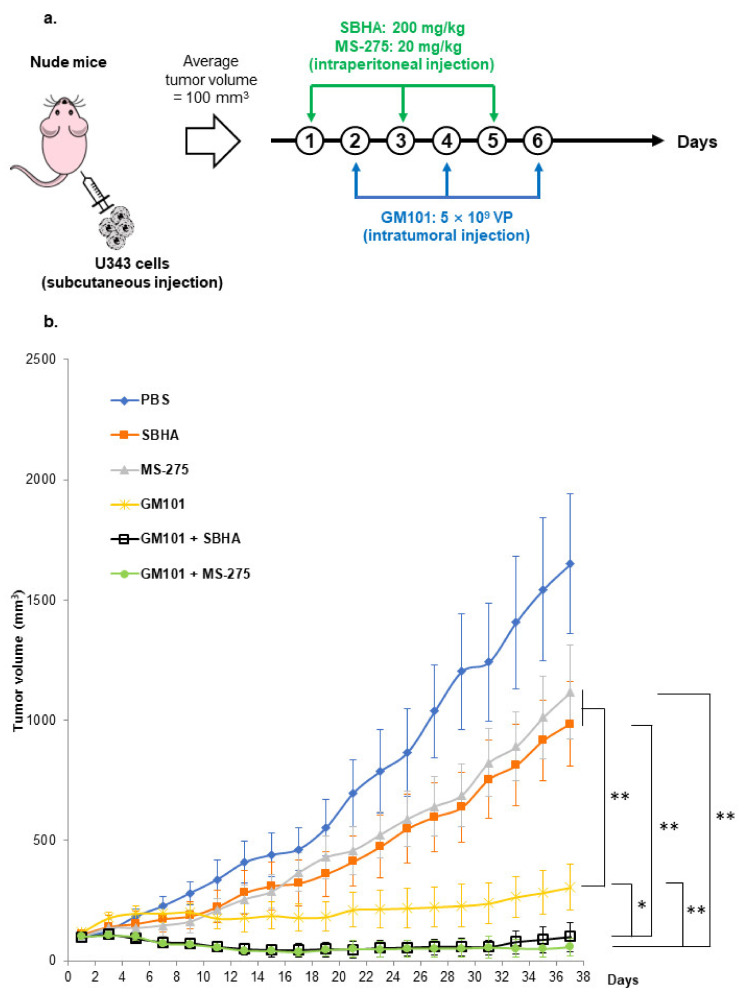
Antitumor effects of GM101 and HDACi combination therapy: (**a**) U343 xenograft tumors were established in nude mice through subcutaneous injection to the abdominal skin. Once the average tumor volume reached 100 mm^3^, tumor-bearing mice were treated with PBS, SBHA (200 mg/kg), MS-275 (20 mg/kg), GM101 (5 × 10^9^ VP), GM101+SBHA, or GM101+MS-275. (**b**) Tumor volume was measured every two days by caliper. Results are expressed as the mean ± SD (each group, *n* = 6). ^*^ *P* < 0.05, ^**^ *P* < 0.01.

To investigate whether HDACi in combination with GM101 can elicit superior tumor growth control than monotherapies in tumor-bearing mice, U343 GBM xenografts were subcutaneously established in nude mice. When the average tumor volume reached 100 mm^3^, mice were randomized and treated with PBS, SBHA, MS-275, GM101, GM101+SBHA, or GM101+MS-275. As shown in Figure 5a, SBHA (200 mg/kg) or MS-275 (20 mg/kg) were administered intraperitoneally every other day for a total of 3 times (day 1, 3, and 5 of treatment), while 5 × 10^9^ VPs of GM101 was intratumorally administered on day 2, 4, and 6 of treatment. As shown in Figure 5b, GM101 monotherapy and combination therapies (GM101+SBHA or GM101+MS-275) induced significant suppression of tumor growth in comparison to SBHA or MS-275 monotherapies at day 37 after the initial treatment (*P* < 0.01). Importantly, GM101+SBHA or GM101+MS-275 combination therapy elicited significantly more potent antitumor effects than either any monotherapy group (*P* < 0.05 or 0.01).

### 3.6. Combination of GM101 with HDACi Increases Viral Accumulation, Apoptotic Cell Death, and Degradation of Extracellular Matrix in Tumor Tissues

**Figure 6:**
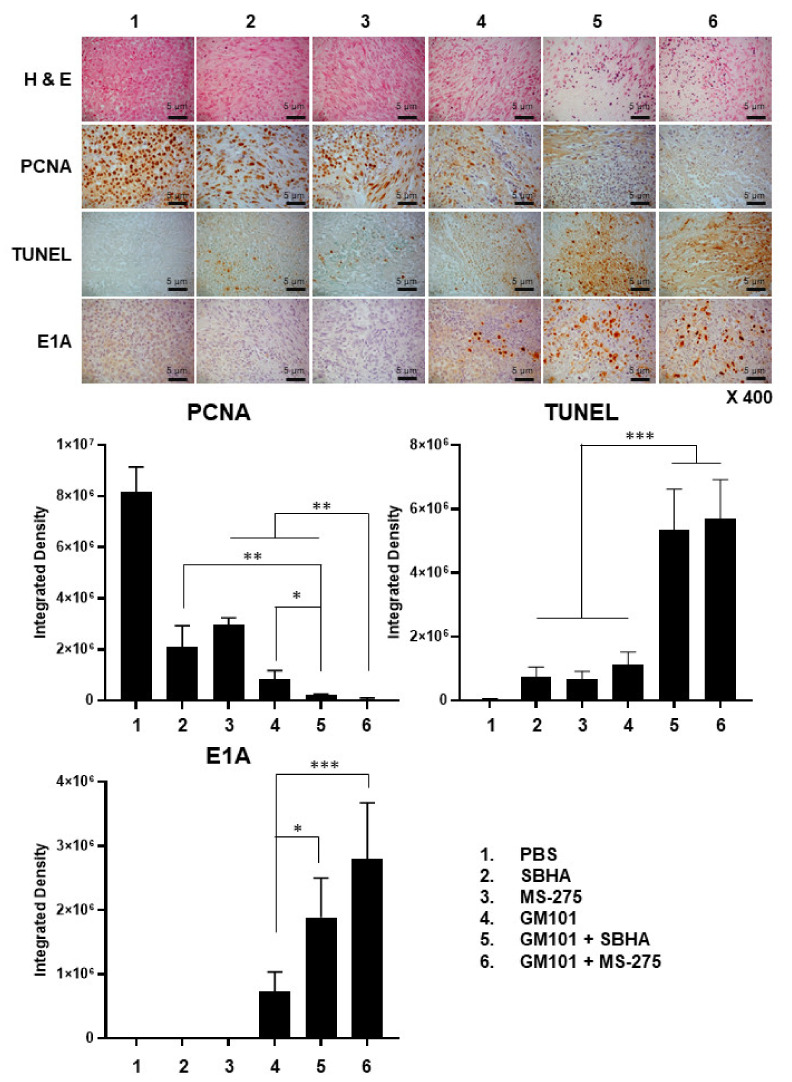
Histological and immunohistochemical analysis of tumor tissues. U343 xenograft tumors were established in nude mice through subcutaneous injection to the abdominal skin. Once the average tumor volume reached 100 mm^3^, tumor-bearing mice were treated with PBS, SBHA (200 mg/kg), MS-275 (20 mg/kg), GM101 (5 × 10^9^ VP), GM101+SBHA, or GM101+MS-275. Tumor tissues were harvested for histological and immunohistochemical analysis on the 9^th^ day after initial treatment. Tumor sections were stained with hematoxylin and eosin (H & E), proliferating cell nuclear antigen (PCNA)-specific antibodies, terminal deoxynucleotidyl transferase dUTP nick end labeling (TUNEL), or Ad E1A-specific antibodies. Representative images are shown. Original magnification: ×400. The scale bar represents 5 µm. Immunohistochemical staining results were semi-quantitatively analyzed using ImageJ analysis software. Data are presented as the mean integrated optical density ± SD (*n* = 3 mice per group; optical density was analyzed from five random fields). ^*^ *P* < 0.05, ^**^ *P* < 0.01, ^***^ *P* < 0.001.

To investigate the mechanisms behind the potent antitumor effect mediated by GM101 and HDACi combination therapies, histological and immunohistochemical analysis of tumor tissues were performed. As shown in Figure 6, H & E and PCNA staining of tumor tissues revealed that all monotherapy groups including GM101, SBHA, or MS-275 induced necrosis and inhibited proliferation of tumor cells compared to the PBS group. Notably, the combination therapy regimens (GM101+SBHA and GM101+MS-275) induced more potent anticancer effects compared with monotherapy groups (*P* < 0.05 or 0.01). TUNEL-positive spots were also detected at the highest level in GM101+SBHA- or GM101+MS-275-treated tumors (*P* < 0.001 versus monotherapy groups), indicating that the combination therapies exert potent antitumor effects via robust induction of apoptosis and reduction of cancer cell proliferation. Importantly, GM101+SBHA- or GM101+MS-275-treated tumor tissues exhibited the highest level of Ad E1A-positive spots in a wider area of the tumor tissue, suggesting HDACi enhanced viral accumulation and dispersion throughout the tumor tissues (*P* < 0.05 or 0.001). Additionally, GM101+SBHA or GM101+MS-275 combination therapy did not elevate liver (ALT/AST) and kidney (BUN/CRE) serum toxicity markers compared to those of PBS-treated mice in A549 tumor-bearing mice (Figure S7), demonstrating that the combination therapy can be administered safely without additional risk of toxicity.

**Figure 7:**
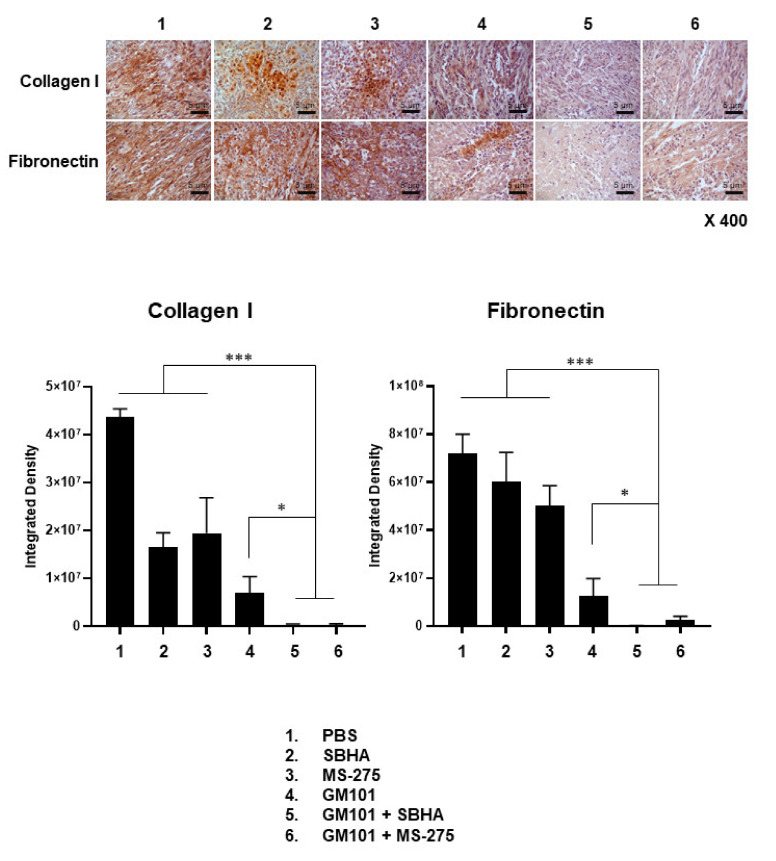
Histological and immunohistochemical analysis of extracellular matrix composition of tumor tissues. U343 xenograft tumors were established in nude mice through subcutaneous injection to the abdominal skin. Once the average tumor volume reached 100 mm^3^, tumor-bearing mice were treated with PBS, SBHA (200 mg/kg), MS-275 (20 mg/kg), GM101 (5 × 10^9^ VP), GM101+SBHA, or GM101+MS-275. Tumor tissues were harvested for histological and immunohistochemical analysis on the 9^th^ day after initial treatment. Tumor sections were stained with collagen I- or fibronectin-specific antibodies. Representative images are shown. Original magnification: ×400. The scale bar represents 5 µm. Immunohistochemical staining results were semi-quantitatively analyzed using ImageJ analysis software. Data are presented as the mean integrated optical density ± SD (*n* = 3 mice per group; optical density was analyzed from five random fields). * *P* < *0.05*, *** *P* < *0.001*.

GM101 has been shown to exert potent tumor ECM degrading effects in our previous reports [27,36]. Thus, we investigated whether the combination therapy regimens (GM101+SBHA and GM101+MS-275) enhanced the ECM degrading property of GM101 in tumor tissues. As shown in Figure 7, GM101 monotherapy led to significant suppression of major tumor ECM components (collagen I and fibronectin) compared to PBS, SBHA, or MS-275 monotherapy groups (*P* < 0.001), demonstrating the potent ECM degrading property of GM101 in tumor tissues. Significantly, GM101+SBHA- or GM101+MS-275-treated tumors exhibited significantly lower levels of major ECM components (collagen I and fibronectin) in comparison to the tumors treated with any of the monotherapy regimens (*P* < 0.05 or *P* < 0.001), suggesting that HDACi can enhance the ECM degrading property of GM101 in tumor tissues. Together, these results demonstrate that the combination of GM101 with HDACi improved the antitumor effect of oncolytic Ad through the enhanced induction of apoptosis, virus accumulation and dispersion, and ECM destruction of tumor tissues.

## 4. Discussion

Despite major advances in cancer therapy, limited intratumoral distribution of anti-cancer therapeutics remains a major challenge toward maximizing the therapeutic potential. In this regard, aberrantly accumulated ECM in the tumor microenvironment has been identified as a key hindrance for the poor dispersion of cancer therapeutics in a tumor mass [36,37]. Additionally, there are therapy-specific obstacles, such as variable CAR expression levels of heterogenic tumor cell population, that attenuate the internalization and dispersion of Ad in tumor tissues. To overcome these challenges, the present report investigated the combination of ECM-degrading oAd (GM101) with HDACi to maximize the intratumoral distribution of virus through upregulation of CAR, and synergistic degradation of ECM and induction of apoptosis.

Our findings demonstrated that both HDACi (SBHA and MS-275) pretreatment elevated CAR expression levels (Figure 2a) and membrane attachment of Ad to the surface of the cancer cell membrane (Figure 2b and Figure S5), in line with several other reports demonstrating that HDACi-treated cells show elevated CAR or αvβ3 integrin expression levels, that enhance cell internalization and transgene expression of Ad [23,38,39,40]. Importantly, our study was the first to demonstrate that HDACi treatments also elevated the expression levels of dynamin 2 and clathrin (Figure S6), which are critical to clathrin-mediated endocytosis of Ad after Ad capsids are docked to CAR and integrin expressed on the cell surface [33]. Inhibition of dynamin 2 and clathrin by small chemicals were shown to markedly attenuate the transduction efficacy of Ad in HDACi-pretreated cells (Figure 3a,b). These findings demonstrated that enhanced Ad transduction in HDACi-pretreated cells was not solely dependent on the upregulation of CAR, but rather achieved via simultaneous elevation of other endocytic factors such as dynamin 2 and clathrin.

Excessive accumulation of tumor ECM forming a dense physical barrier against drug penetration is another major hindrance to maximizing intratumoral accumulation and dispersion of oncolytic adenovirus. In this regard, HDACi (SBHA or MS-275) and GM101 as a monotherapy promotes the degradation of a major ECM component, collagen I (Figure 7). These findings are in line with previous reports, demonstrating that GM101 or HDACi inhibit intratumoral ECM synthesis via upregulation of matrix metalloproteinases [36] or inhibition of TGF-β1 signaling pathways [41], respectively. Importantly, the combination of GM101 and HDACi (GM101+SBHA or GM101+MS-275) led to a greater level of ECM (collagen I and fibronectin) degradation than those achieved by any monotherapies, suggesting that GM101 and HDACi could enhance the ECM degrading effect in tumor tissue. The potent ECM degrading effect by the combination therapy (GM101+SBHA or GM101+MS-275) positively correlated with increased viral dispersion and accumulation in tumor tissues (Figure 6), demonstrating that robust ECM degradation can maximize intratumoral distribution of oncolytic Ad. Despite these promising results in the subcutaneous GBM tumor model, it should be noted that subcutaneous and orthotopic GBM tumor models exhibit several differences in disease progression and characteristics [42,43,44,45,46]. Intriguingly, subcutaneous GBM tumors have been reported to show higher density of fibroblast-like cells, type I collagen, and fibrillar collagen than the orthotopic counterpart [47], which may arise due to ECM composition in normal brain tissue being highly specialized and distinct from that of any other tissues in the body [48]. It should also be noted that both subcutaneous and orthotopic xenograft tumor models derived from a human cancer cell line cannot fully recapitulate the desmoplastic tumor microenvironment of clinical tumors due to many of the integral oncogenesis process along with key environmental factors (e.g., cancer-associated fibroblasts) being absent or of murine origin, thus exhibiting different characteristics to those observed in patient tumor samples [42,49,50,51,52]. As these microenvironmental factors play an integral role in multiple biological processes such as desmoplasia and progression of clinical tumors, a more in-depth evaluation of the combinatory effect of GM101 and HDACi will be conducted in patient-derived xenograft models or those utilizing patient-derived tumor explants to obtain more clinically relevant data in a future study.

Other than an aberrantly dense layer of ECM, a high level of interstitial fluid pressure in the tumor has been reported to be a major hindrance toward drug dispersion [53,54]. To this end, several reports have identified robust induction of apoptosis or decreased tumor cell density/mass in solid tumors to abrogate this prohibitive effect on drug dispersion by attenuating the interstitial fluid pressure and generating loose spaces to facilitate drug diffusion [25,55,56]. Additionally, the apoptotic bodies can also function as vesicles that aid in spreading viral progenies to peripheral tumor cells [25,56]. In line with these previous reports, a positive correlation between robust apoptosis and virus dispersion in tumor tissues was also observed: in detail, the combination therapy (GM101+SBHA or GM101+MS-275) with the highest level of apoptosis also exhibited the highest level of virus dispersion in tumor tissues (Figure 6). Further, apoptosis in tumor tissues by the combination therapy (GM101+SBHA or GM101+MS-275) was more potent than the summation of the respective monotherapy (e.g., integrated density of TUNEL staining in GM101+SBHA combination therapy is greater than the summation of SBHA and GM101 monotherapies), suggesting a significantly increased induction of apoptosis.

Collectively, these findings demonstrate that through the intratumoral dispersion of the oAd via multifaceted mechanisms (increased internalization into cancer cells via upregulating key factors in CAR-dependent or dynamin-dependent clathrin endocytic pathways, ECM destruction, and induction of apoptosis), the combination of ECM-degrading oAd and HDACi is an effective strategy to maximize the potent antitumor efficacy of each monotherapy.

## Data Availability

All data generated or analyzed during this work are included in this article.

## Supplementary Materials

The following are available online at https://www.mdpi.com/article/10.3390/cells10112811/s1, Figure S1: Schematic representation of GM101, Figure S2: Brightfield images of Figure 1, Figure S3: Adenoviral transgene expression after treatment of HDACi TSA and Valproic acid, Figure S4: Raw data of Figure 2a, Figure S5: Cell attachment of Ad to HDACi pretreated cells, Figure S6: Expression of dynamin-2 and clathrin after treatment with HDACi SBHA and MS-275, Figure S7: Measurement of serum AST, ALT, BUN and CRE of A549 tumor-bearing mice after treatment with PBS, GM101+SBHA, and GM101+MS-275
