# Supplementary figures and images for: GM101 in Combination with Histone Deacetylase Inhibitor Enhances Anti-Tumor Effects in Desmoplastic Microenvironment
Source: Cells. 2021 Oct 20;10(11):2811. doi: 10.3390/cells10112811 (PMC8616263; doi:10.3390/cells10112811)

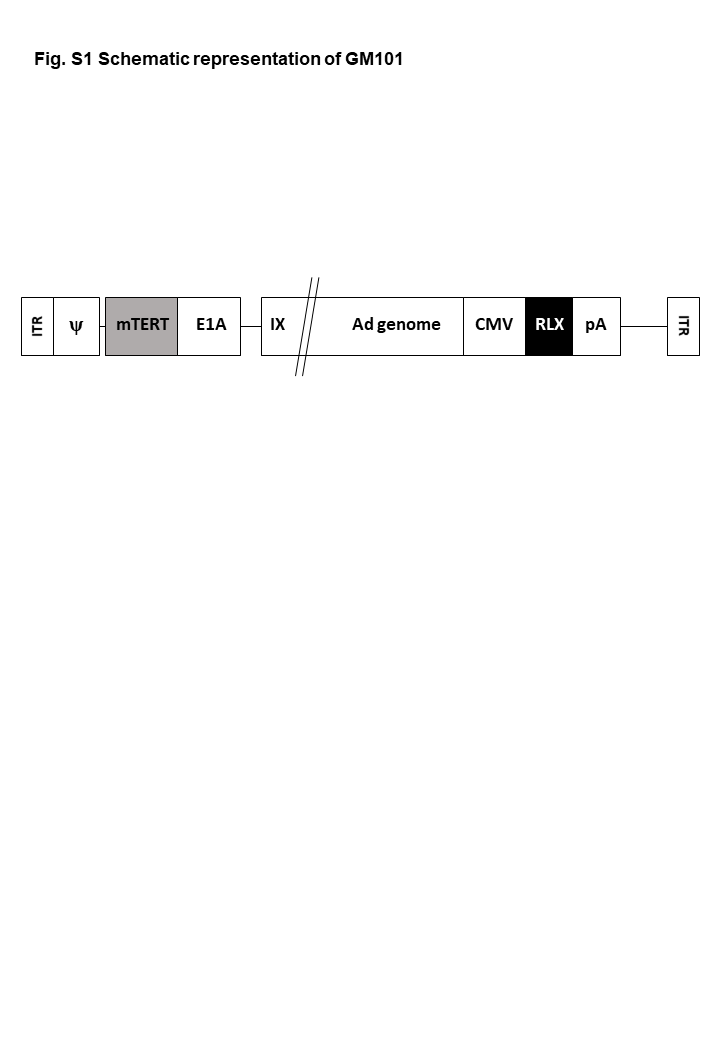

Supplement: Supplementary file 1 [file cells-10-02811-s001.zip › cells-1334789-supplementary PUB/Figure S1.TIF]

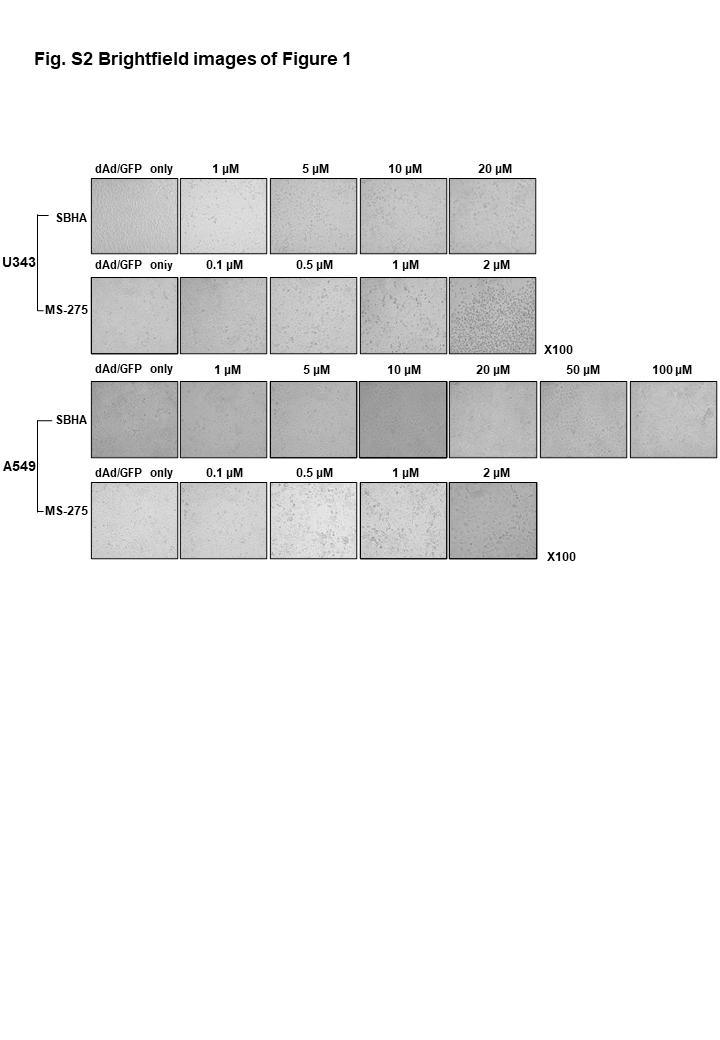

Supplement: Supplementary file 1 [file cells-10-02811-s001.zip › cells-1334789-supplementary PUB/Figure S2.TIF]

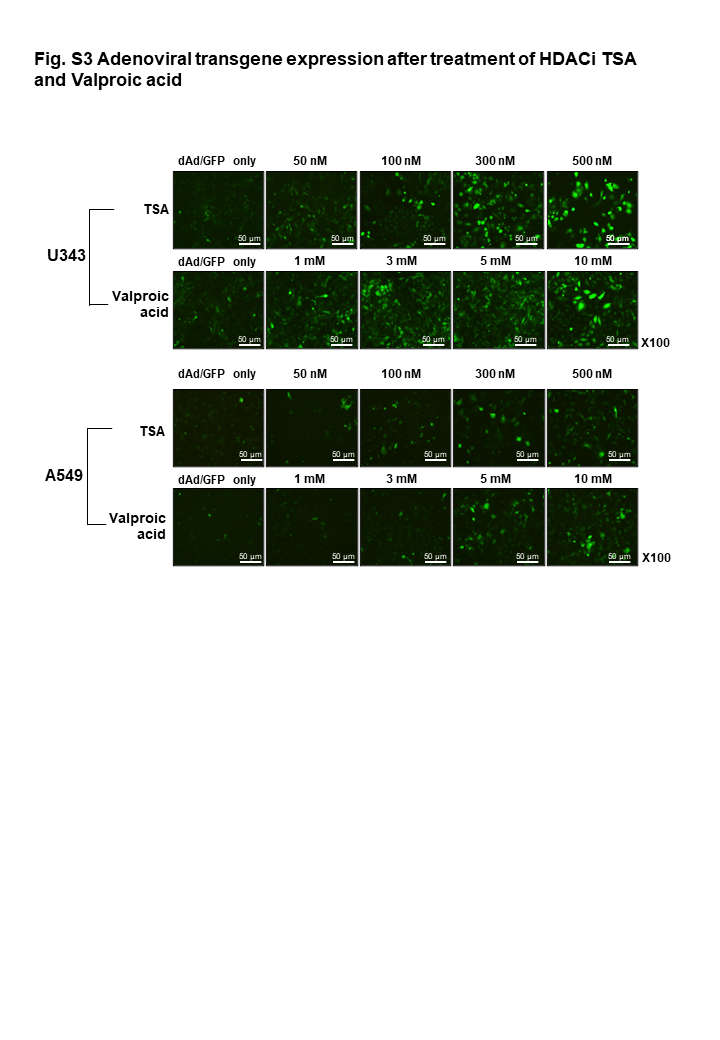

Supplement: Supplementary file 1 [file cells-10-02811-s001.zip › cells-1334789-supplementary PUB/Figure S3.TIF]

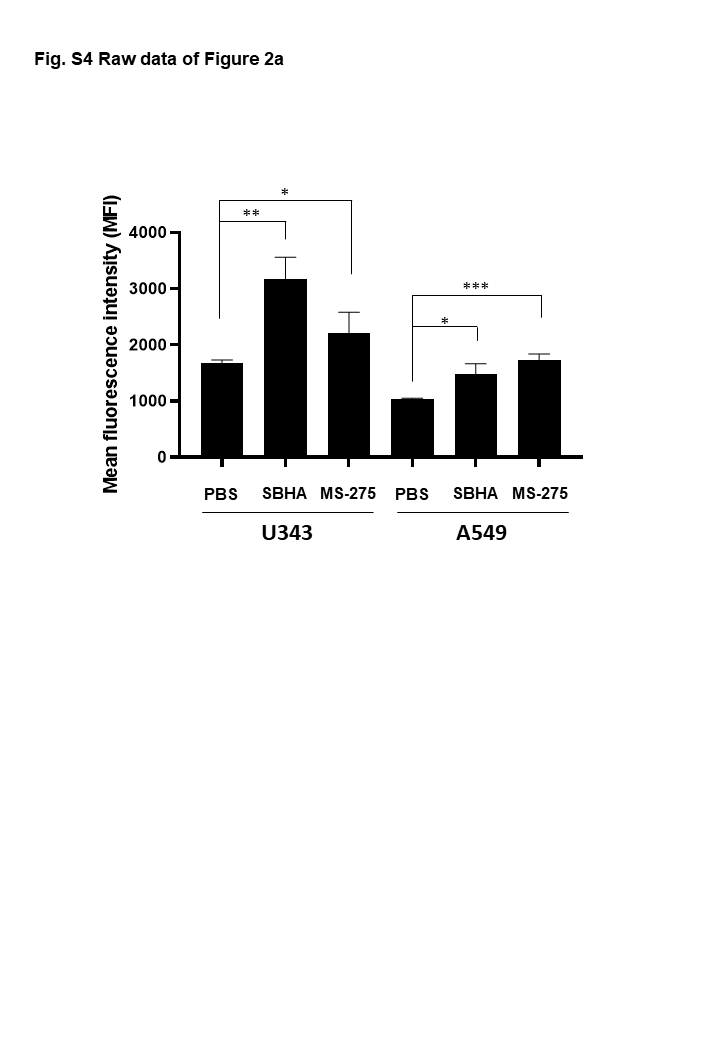

Supplement: Supplementary file 1 [file cells-10-02811-s001.zip › cells-1334789-supplementary PUB/Figure S4.TIF]

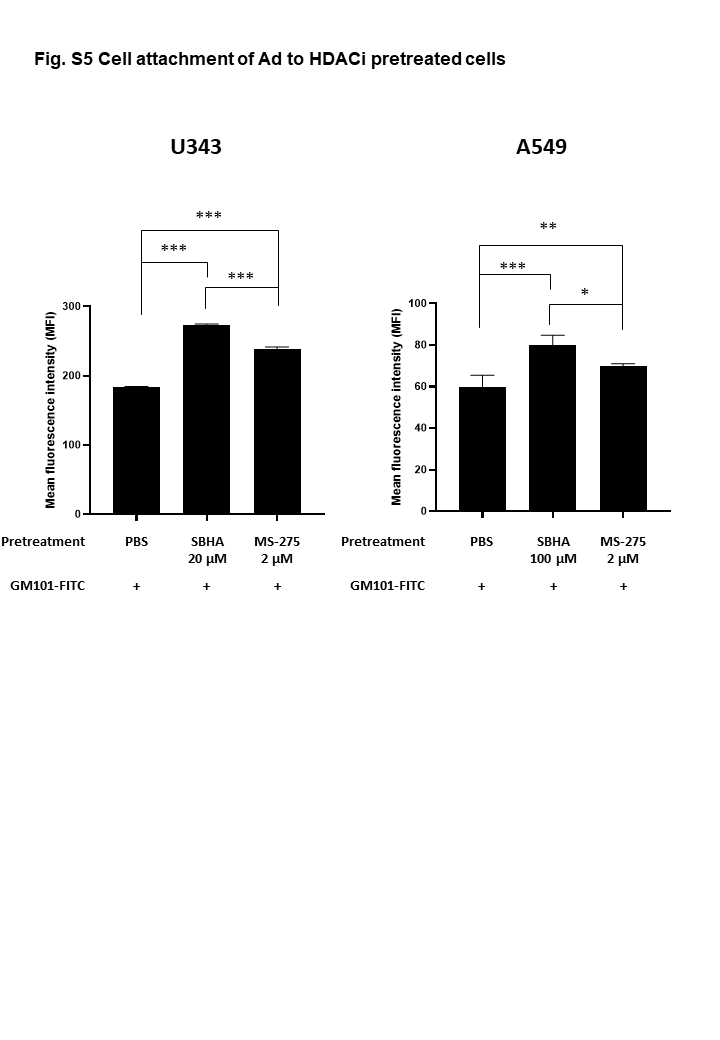

Supplement: Supplementary file 1 [file cells-10-02811-s001.zip › cells-1334789-supplementary PUB/Figure S5.TIF]

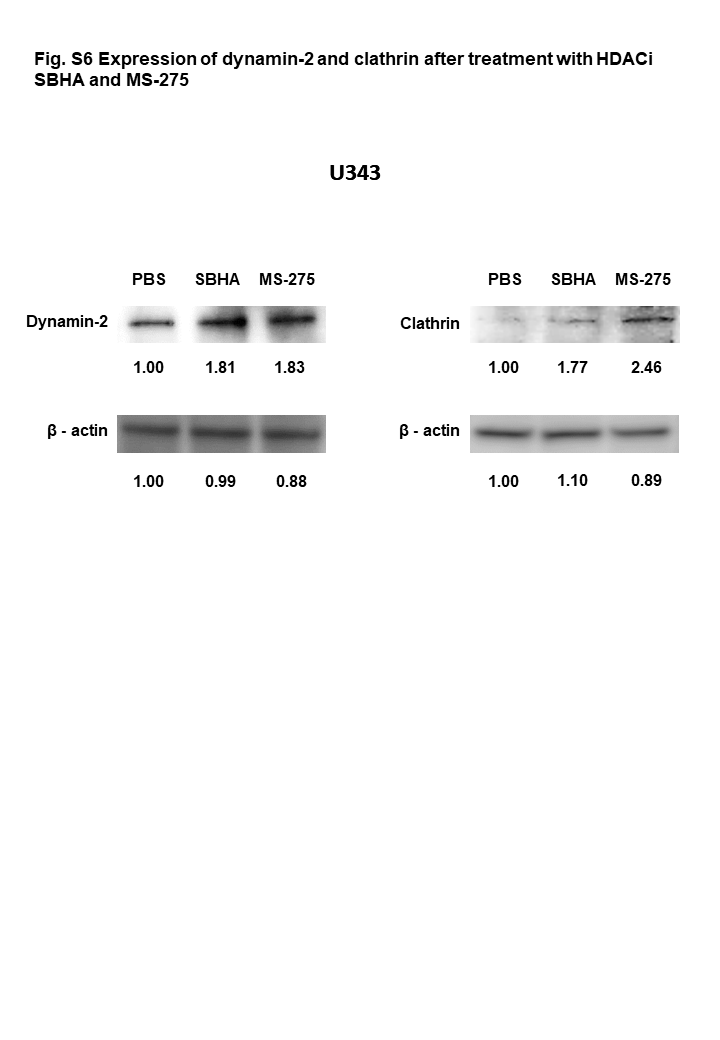

Supplement: Supplementary file 1 [file cells-10-02811-s001.zip › cells-1334789-supplementary PUB/Figure S6.TIF]

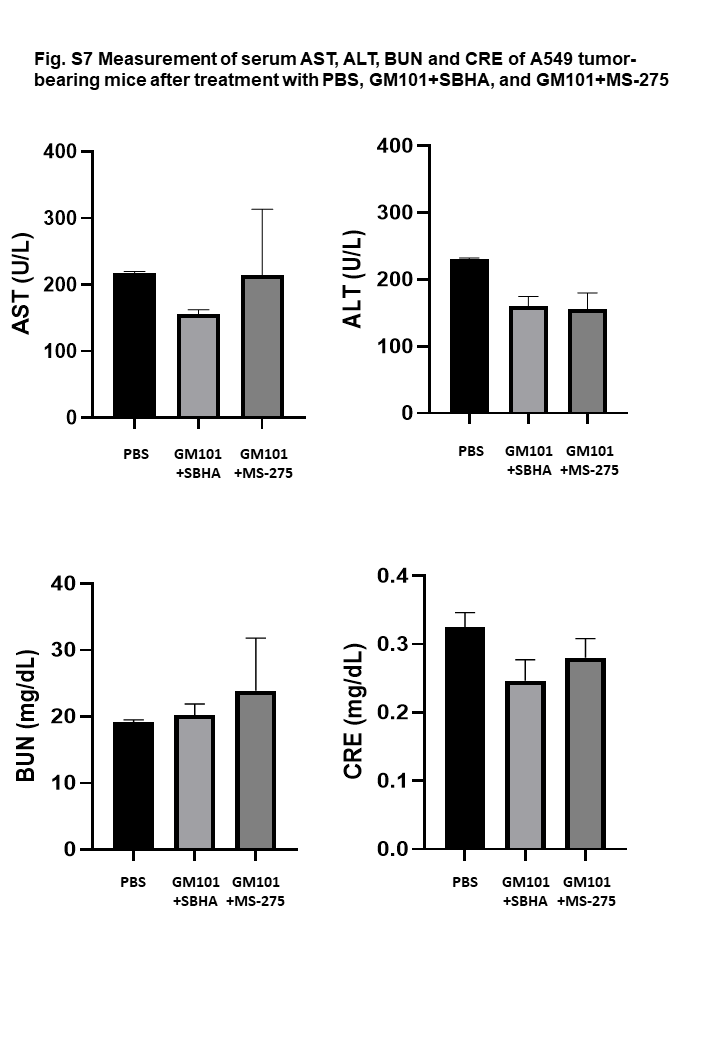

Supplement: Supplementary file 1 [file cells-10-02811-s001.zip › cells-1334789-supplementary PUB/Figure S7.TIF]
